# Regulatory patterns of Chinese patent medicine for lipid metabolism disorders in patients with type 2 diabetes mellitus complicated by ischemic stroke: A systematic review and network meta-analysis

**DOI:** 10.1097/MD.0000000000035050

**Published:** 2024-05-17

**Authors:** Zhenkai Lu, Xuming Zhang, Yanming Xie

**Affiliations:** a Institute of Basic Research in Clinical Medicine, China Academy of Chinese Medical Sciences, Beijing, China.

**Keywords:** Chinese patent medicine, dyslipidemia, ischemic stroke, network meta-analysis, T2DM

## Abstract

**Background:**

To investigate the regulatory patterns of Chinese patent medicine (CPM) interventions on lipid metabolism disorders in patients with type 2 diabetes mellitus (T2DM) complicated by ischemic stroke.

**Methods:**

Two researchers independently searched 8 major databases and created a comprehensive database containing all randomized controlled trials (RCTs) that investigated the application of “blood-activating and stasis-removing” CPM in the treatment of stroke combined with T2DM until October 1, 2022. The collected data were compiled and organized in Excel. Quality assessment was performed using the Cochrane 5.3 bias risk assessment tool, and the network meta-analysis was conducted using R software.

**Results:**

A total of 12 articles were included in the final analysis, covering 4 types of CPM: Naoxintong Capsules (NXT), Tongmai Jiangtang Capsules, Tongxinluo Capsules (TXL), and Yindan Xinnaotong Soft Capsules. Among these, CPM formulations containing herbs with blood-activating and stasis-removing properties were the most commonly used. The results of the network meta-analysis are as follows: (1) the combination of 3 CPM formulations showed superior efficacy in improving total cholesterol levels compared to conventional Western medicine treatment (CT). In particular, Yindan Xinnaotong Soft Capsules + CT (surface under the cumulative ranking curve [SUCRA] = 97.24%) demonstrated the highest efficacy, followed by NXT + CT (SUCRA = 66.23%), and then TXL + CT (SUCRA = 55.16%). (2) TXL + CT treatment exhibited the most promising efficacy in improving triglyceride levels (*P* < .05), while the effects of the other 3 CPM formulations were not statistically significant. (3) In terms of improving low-density lipoprotein levels, NXT + CT (SUCRA = 82.27%) showed better efficacy than TXL + CT (SUCRA = 73.99%), while the effects of the other 2 CPM formulations were not statistically significant. (4) The combination of CPM formulations and CT resulted in a lower incidence of adverse reactions compared to CT (*P* < .05).

**Conclusion:**

The treatment of patients with T2DM complicated by ischemic stroke commonly involved the use of “blood-activating and stasis-removing” herbal medicines. These herbal medicines have shown effectiveness in regulating patients’ blood lipid levels. However, it is crucial to acknowledge that the analysis was influenced by variations in the number and quality of RCTs involving different CPM formulations. Therefore, additional validation through large-scale, high-quality RCT studies is required.

## 1. Background

The World Health Organization defines “comorbidity” as the coexistence of 2 or more health conditions that necessitate ongoing and diverse treatments and can mutually influence each other. In China, the prevalence of chronic diseases is 69.13%, and the comorbidity rate is 43.65%. Among these, multiple diseases commonly coexist among elderly patients with type 2 diabetes.^[1]^ Recent data from the British Medical Journal indicates that the prevalence of comorbidity in high-income countries is mainly influenced by age. The proportion of individuals with 2 or more diseases is steadily increasing due to changes in the population structure, and this trend is expected to continue.^[2]^ In the treatment of comorbidities, Western medicine often concentrates on individual diseases, overlooking the interconnections between comorbid conditions, which can result in suboptimal clinical outcomes.

Dyslipidemia acts as a connection between type 2 diabetes mellitus (T2DM) and ischemic stroke. About 42% of T2DM patients experience dyslipidemia.^[3]^ This condition can lead to insulin resistance, further disrupting blood glucose regulation and exacerbating brain damage in individuals with ischemic stroke, significantly impacting disease prognosis.^[4]^ Research studies have shown that reducing serum levels of total cholesterol, triglycerides, and low-density lipoprotein can significantly reduce the incidence of microvascular and macrovascular complications in T2DM, playing a crucial role in preventing and treating ischemic stroke.^[5,6]^

Chinese patent medicine (CPM) incorporates unique characteristics, including a “holistic perspective” and “pattern-based treatment,” allowing for the examination of correlations between apparently unrelated comorbidities using CPM patterns. This approach holds significant value and should be promoted and implemented in clinical practice. Nevertheless, the effectiveness of different CPM formulations varies, and comprehensive cross-comparisons are presently inadequate. To bridge this research gap, our study performed a network meta-analysis comparing the impacts of diverse CPM formulations on the enhancement of total cholesterol, triglycerides, and low-density lipoprotein levels in patients with T2DM complicated by ischemic stroke. Our objective is to present unbiased and rigorous evidence grounded in the principles of evidence-based medicine.

## 2. Methods

The protocol was registered with the registration number CRD42023401605.

### 2.1. Search strategy

Two researchers independently conducted a literature search using keywords such as “diabetes mellitus,” “stroke,” “granules,” “randomized controlled trial,” “type 2 diabetes mellitus,” “Chinese patent medicine,” and “randomized controlled trial.” The search covered 8 prominent databases, including PubMed, Web of Science, Embase, Cochrane, CNKI, Wanfang Data, VIP Database, and China Biomedical Literature Database. The search spanned from the inception of each database until October 1, 2022, and encompassed published literature. Distinct search strategies were employed for each database, employing a combination of different search terms. Table 1 displays the search strategies employed for PubMed.

**Table 1:** Pubmed search strategy.

No	Search strategy
#1	((diabetes mellitus, type 2[MeSH Terms]) OR (Noninsulin Dependent Diabetes Mellitus[Title/Abstract])) OR (NIDDM[Title/Abstract]) OR (T2DM[Title/Abstract])
#2	((((((((((((((stroke[MeSH Terms]) OR (apoplexy[Title/Abstract])) OR (apoplexia[Title/Abstract])) OR (brain infarction[Title/Abstract])) OR (cerebral infarction[Title/Abstract])) OR (cerebrovascular occlusion[Title/Abstract])) OR (brain ischemia[Title/Abstract])) OR (cerebral embolism[Title/Abstract])) OR (brain embolism[Title/Abstract])) OR (intracranial embolism[Title/Abstract])) OR (cerebral thrombosis[Title/Abstract]))
#3	((((((((Pill) OR (Granules)) OR (Capsule)) OR (Tablets)) OR (proprietary Chinese medicine)) OR (traditional Chinese medicine)) OR (Chinese patent medicine)) OR (powder)) OR (oral liquid)
#4	((((((randomized controlled trial[Title/Abstract]) OR (clinical trial[Title/Abstract])) OR (randomized[Title/Abstract])) OR (controlled[Title/Abstract])) OR (trial[Title/Abstract])) OR (random[Title/Abstract])) OR (double-blind[Title/Abstract])
#5	#1 AND #2 AND #3 AND #4

### 2.2. Inclusion and exclusion criteria

Inclusion criteria: (1) types of studies: randomized controlled trials (RCTs). (2) Types of participants: patients with T2DM^[7]^ and stroke.^[8]^ (3) Type of interventions: oral CPM combined with conventional western medicine treatment (CT), including low-salt and low-fat diet, blood glucose control, blood lipid regulation, improvement of blood coagulation status, etc, in the treatment group versus CT alone in the control group, with no restrictions on the duration of treatment. (4) Prescription forms of CPM: CPMs are market-approved, come with comprehensive instruction manuals, and are intended for oral administration. (5) Types of outcomes: low-density lipoprotein (LDL), triglyceride (TG), total cholesterol (TC); (6) patients with stable vital signs and good compliance.

Exclusion criteria: (1) types of studies: reviews, social reviews, case reports, case series analyses, cell experiments, animal experiments, etc. (2) Types of participants: Patients with severe complications, such as liver or kidney failure, gastrointestinal bleeding, etc., were excluded from the study. (3) Types of interventions: non-oral CPM, including enemas, intravenous injections, plasters, foot baths, etc; non-clinically prescribed decoctions; use of CPMs in the control group. (4) Types of outcomes: absence of primary or secondary outcomes. (5) To ensure the stability of data analysis as much as possible, studies with fewer than 2 publications were excluded.

### 2.3. Data extraction and quality assessment

Upon completing the literature search, we utilized the NoteExpress literature management tool to eliminate duplicate entries from the literature gathered from the 8 major databases. Any remaining duplicates were manually removed. The final selection of literature was meticulously determined based on the specified inclusion and exclusion criteria. Baseline data, including the first author, publication year, literature title, number of participants, intervention measures, and outcome indicators, were independently extracted from the chosen literature by 2 researchers, Lu Zhenkai and Zhang Xuming. The collected data underwent cross-verification. Additionally, the quality of the included studies was assessed independently by the same 2 authors using the Cochrane 5.3 bias risk assessment tool. In the event of any discrepancies, they were resolved through discussion and consultation with Xie Yanming.

### 2.4. Statistical methods

A Bayesian network meta-analysis was conducted using the R software and the R language package BUGSnet to calculate the relative effect value. The analysis employed the Bayesian method, and the selection of the effect model was based on the deviation information criterion (DIC). The DIC value served as an indicator of how well the sample size fitted the model, with a smaller DIC value indicating better fit. The mean differences for TG, TC, and LDL, which are continuous variables, were calculated. The 95% confidence intervals were established, and a significance level of *P* < .05 was considered statistically significant for indicating differences. The ranking of outcome indicators following the intervention of each CPM was evaluated using the surface under the cumulative ranking curve (SUCRA). A higher SUCRA value indicates a better efficacy of the CPMs for that specific outcome.

The Bayesian network meta-analysis was conducted using the nma.run() function. The network diagram was generated using the net.plot() function. The treatment ranking result diagram was created using the nma.rank() function. The SUCRA and rankogram were employed to predict the ranking probability of each intervention. The Heat Map was generated using the nma.league() function.

The quality of evidence was assessed using the GRADE methodology. Factors contributing to the downgrading of evidence included study limitations, inconsistency, indirectness, imprecision, and publication bias. The evidence quality was rated on a scale from A to D.

## 3. Results

### 3.1. Study identification

**Figure 1.**
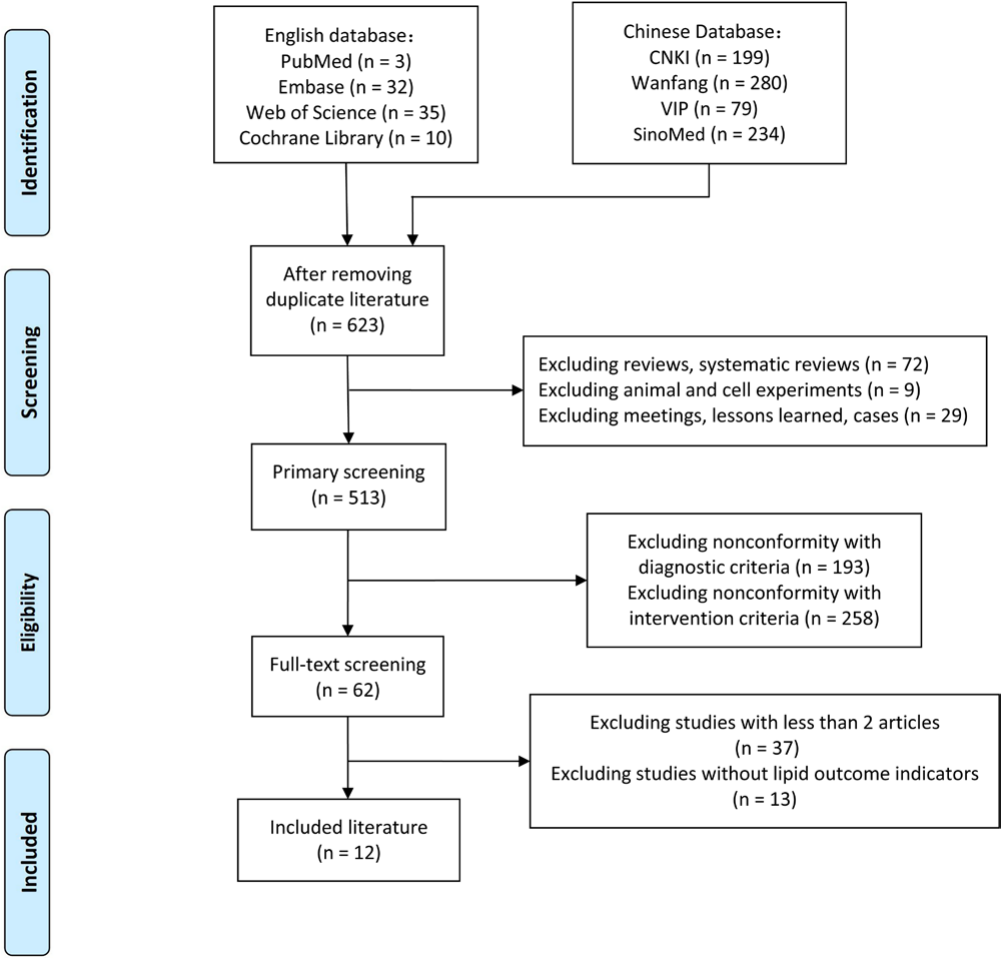
Screening flow chart.

In this study, a total of 80 English articles and 792 Chinese articles were initially identified through the screening process. Articles that did not meet the criteria were excluded based on a review of their titles and abstracts. Additionally, CPMs with <2 relevant articles were excluded. Ultimately, 12 RCTs^[9–20]^ were included for analysis. A detailed description of the screening process can be found in Figure 1.

### 3.2. Characteristics of included studies

A total of 12 articles were included in the analysis, involving a combined total of 1187 patients across 4 intervention groups. Among them, 5 articles^[9–12,18]^ were related to Tongxinluo Capsule (TXL), with a sample size of 443 cases. 2 articles^[16,17]^ were related to Tongmai Jiangtang Capsules (TMJT), with a sample size of 149 cases. 3 articles^[15,19,20]^ were related to Naoxintong Capsule (NXT), with a sample size of 345 cases. Lastly, 2 articles^[13,14]^ were related to Yindan Xinnaotong Soft Capsules (YDXNT), with a sample size of 250 cases. The basic characteristics of the included studies can be found in Table 2.

**Table 2:** Basic characteristic table.

Study	Experiment/control	Samples (No.)	Age (years)	Gender (male/female)	Course of disease (years)	Frequency/dose and course of treatment	Outcomes
Yuan, M. (2022)^[9]^	TXL + CT	34	66.06 ± 9.83	18/16 16/18	N	TID	①②③
	CT	34	67.94 ± 11.51				
Lu, Y. (2014)^[10]^	TXL + CT	32	N	18/14	N	TID, 4 weeks	①②③
	CT	26		16/10			
Dong, R. (2015)^[11]^	TXL + CT	64	75.3 ± 1.6	36/28	5.6 ± 1.9	TID, 4 weeks	①②③
	CT	52	75.5 ± 1.9	32/20	5.9 ± 2.1		
Sun, B. (2019)^[12]^	TXL + CT	49	51.38 ± 6.12	25/24	6.78 ± 0.91	TID	①②③
	CT	49	52.88 ± 6.45	27/22	7.11 ± 1.11		
Zhao, J. (2016)^[13]^	YDXNT + CT	75	60.6 ± 7.5	51/24	N	TID	①②③
	CT	75	58.8 ± 8.7	48/27			
Xiao, D. (2012)^[14]^	YDXNT + CT	70	62.4 ± 7.3	38/32	N	TID, 1 month	③
	CT	30	61.44 ± 8.3	17/13			
Wang, L. (2010)^[15]^	NXT + CT	120	61	72/48	N	TID, 1 month	①②③
	CT	105	60.5	70/35			
An, X. (2018)^[16]^	TMJT + CT	41	60.7 ± 5.8	26/15	7.7 ± 3.1	TID, 12 weeks	①②③
	CT	41	60.6 ± 5.5	25/16	7.5 ± 3.5		
Chen, X. (2016)^[17]^	TMJT + CT	33	60.7 ± 9.7	19/14	6.3 ± 2.4	TID, 3 months	①②③
	CT	34	62.4 ± 8.9	22/12	7.6 ± 3.7		
Wu, M. (2015)^[18]^	TXL + CT	52	63.6 ± 10.7	35/17	N	TID, 4 weeks	①②③
	CT	51	65.7 ± 11.4	31/20			
Zhong, J. (2011)^[19]^	NXT + CT	20	69. 96 ± 9. 21	12/8	8.9 ± 2.4	TID, 4 weeks	①②
	CT	20	71. 84 ± 7. 23	11/9	9.5 ± 2.6		
Fu, Q. (2017)^[20]^	NXT + CT	40	66.1 ± 4.9	22/18	8.3 ± 3.3	TID, 1 month	①②③
	CT	40	66.4 ± 5.1	24/16	8.3 ± 3.6		

*Note*: ① = TC; ② = TG; ③ = LDL; N = no clear.

### 3.3. Quality assessment

According to the Cochrane 5.3 bias risk assessment tool, 2 researchers independently conducted quality evaluations of the included studies. After cross-validation, the following conclusions were drawn: Among the 12 included articles, 4 articles^[9,11,17,18]^ adequately described the generation of random sequences.

**Figure 2.**
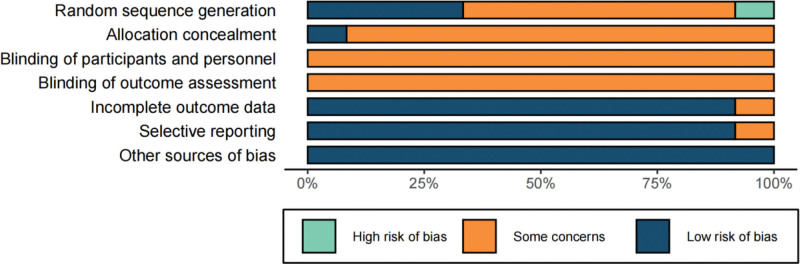
Bias risk assessment diagram.

Seven articles^[10,13–16,19,20]^ mentioned the term “random” without specifying the actual method used for grouping. One article^[18]^ explicitly mentioned the implementation of allocation concealment. None of the articles clearly stated whether outcome assessment and blinding of participants were implemented. 1 article^[10]^ did not specify the presence of reporting bias. The data integrity of the results was ensured, and all studies had no other risk of bias (Fig. 2).

### 3.4. Network evidence structure and model selection

**Figure 3.**
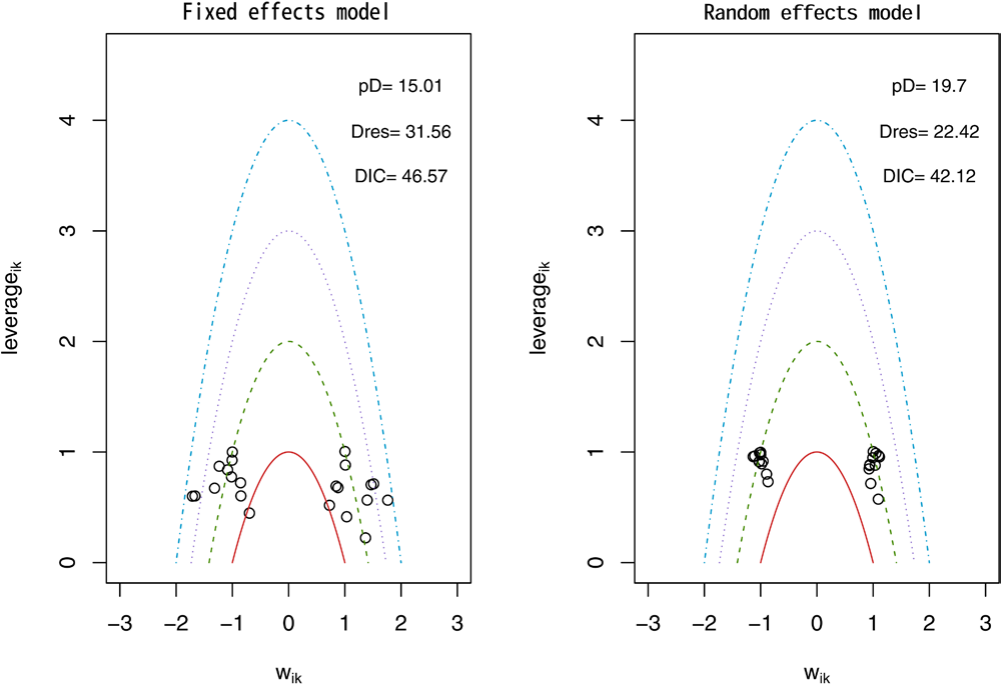
Total cholesterol model selection plot.

**Figure 4.**
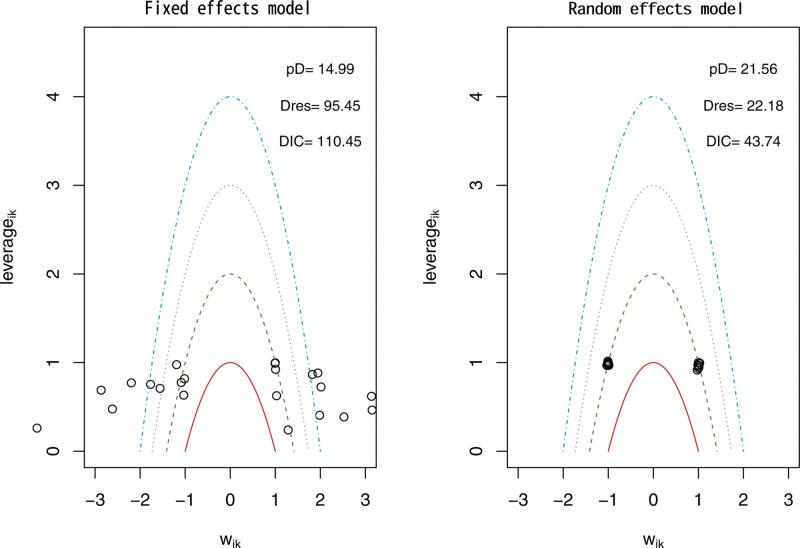
Triglyceride model selection plot.

**Figure 5.**
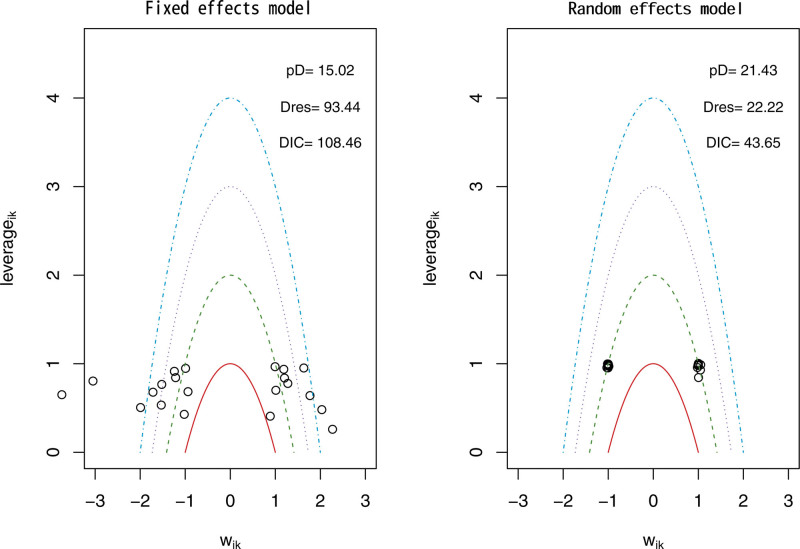
LDL model selection plot.

The network evidence framework consisted of the 12 included (RCTs across 4 intervention groups). To determine the best model fit, the DIC was utilized, with a smaller DIC value indicating better model fit. For the analysis of TC, TG, and LDL, the random-effects model was employed using the network meta-analysis approach. The DIC values were 42.12 for TC (DIC = 42.12 < 46.57, Fig. 3), 43.74 for TG (DIC = 43.74 < 110.45, Fig. 4), and 43.65 for LDL (DIC = 43.65 < 108.46, Fig. 5).

### 3.5. Total cholesterol

**Figure 6.**
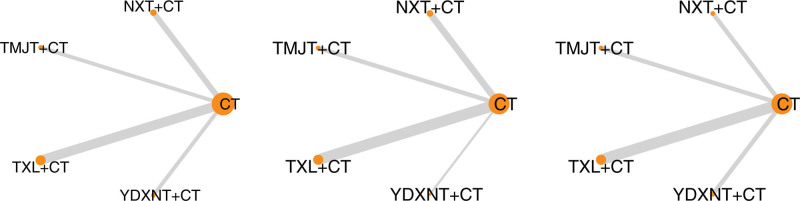
Evidence network diagram. Note: The thickness of the line segment in the figure represents the number of studies in which the 2 interventions can be directly compared, and the size of the point represents the total sample size of the intervention included in the study.

A total of 11 RCTs^[9–13,15–20]^ were included in the analysis, covering the 4 CPM interventions, to assess the improvement of total cholesterol levels in patients with T2DM complicated by ischemic stroke. Among these studies, the comparison of TXL combined with CT versus CT had the highest number of studies (5^[9–12,18]^) and the largest overall sample size (443 cases) (Fig. 6).

**Figure 7.**
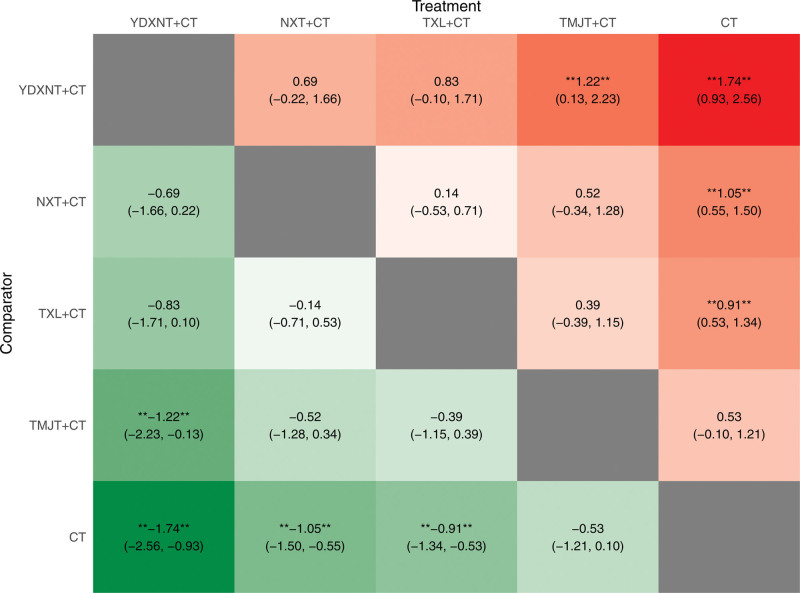
Heat map of TC. Note: * showed that there was a significant difference in the comparison results (*P* < .05).

The results (Fig. 7) demonstrated that compared to CT, the interventions of YDXNT + CT, NXT + CT, and TXL + CT showed statistically significant improvements in total cholesterol levels (*P* < .05). However, there was no significant difference observed for TMJT combined with CT (*P* > .05).

**Figure 8.**
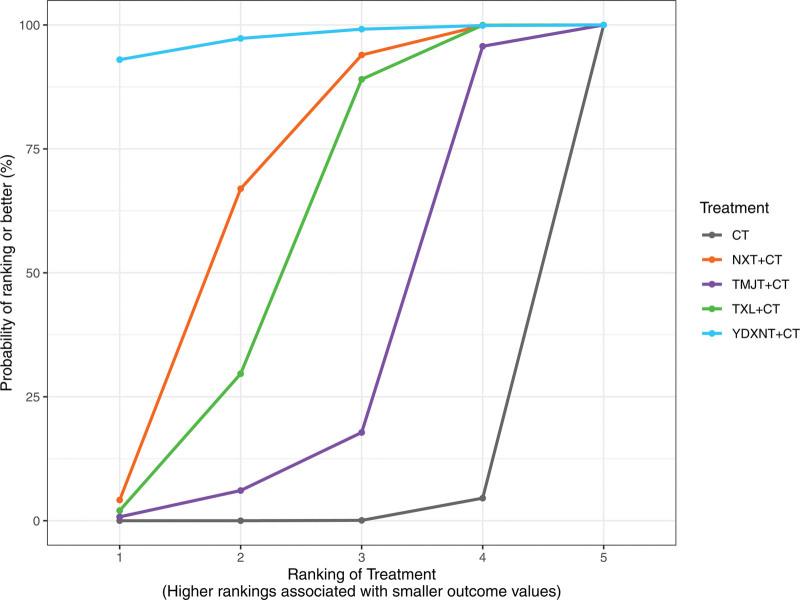
SUCRA ranking diagram of TC.

Based on the surface under the SUCRA probabilities ranking, the interventions were ranked as follows: YDXNT + CT (SUCRA = 97.24%) > NXT + CT (SUCRA = 66.23%) > TXL + CT (SUCRA = 55.16%) > TMJT + CT (SUCRA = 30.21%) > CT alone (SUCRA = 1.15%). This indicates that YDXNT + CT may be the most effective intervention for improving total cholesterol levels in patients with T2DM complicated by ischemic stroke (Fig. 8).

### 3.6. Triglyceride

A total of 11 RCTs^[9–13,15–20]^ investigated the impact of all 4 CPM interventions on improving TG levels in patients with T2DM complicated by ischemic stroke. The network meta-analysis of the 4 direct comparisons revealed that TXL combined with CT had the highest number of studies (5^[9–12,18]^) and the largest overall sample size (443 cases) compared to CT (Fig. 6).

**Figure 9.**
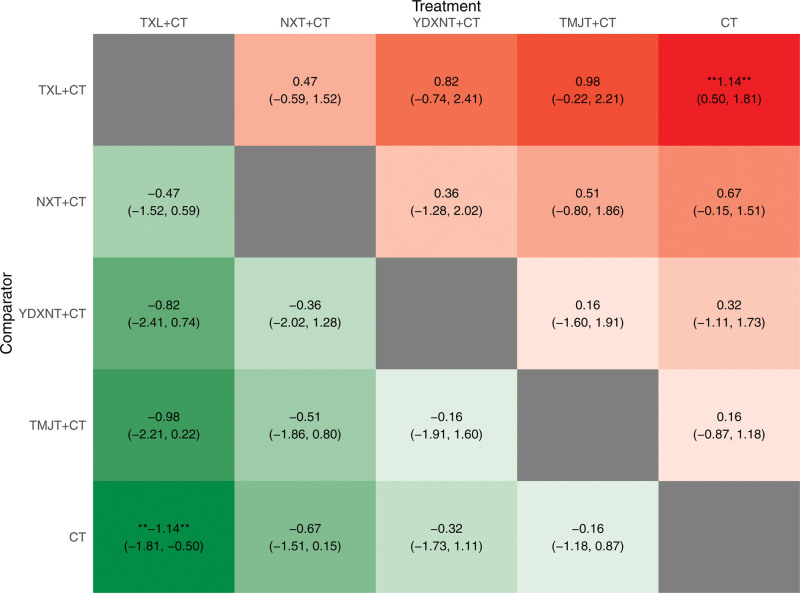
Heat map of TG.

The results demonstrated that only TXL combined with CT significantly improved TG levels compared to CT, with a statistically significant difference (*P* < .05). However, there was no significant difference in the improvement of TG levels between the other 3 CPM interventions combined with CT and CT (*P* > .05), as depicted in Figure 9.

**Figure 10.**
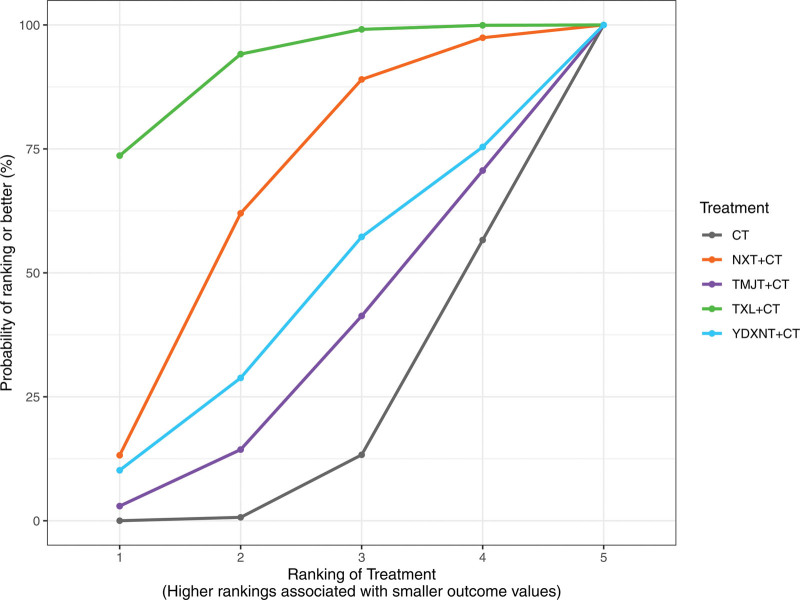
SUCRA ranking diagram of TG.

Based on the surface under the SUCRA probabilities ranking, the interventions were ranked as follows: TXL + CT (SUCRA = 91.72%) > NXT + CT (SUCRA = 65.45%) > YDXNT + CT (SUCRA = 42.86%) > TMJT + CT (SUCRA = 32.16%) > CT alone (SUCRA = 17.81%). These results suggest that TXL + CT may be the optimal intervention for improving TG levels in patients with T2DM complicated by ischemic stroke, as depicted in Figure 10.

### 3.7. Low-density lipoprotein

A total of 11 RCTs^[9–18,20]^ investigated the improvement of LDL levels in patients with T2DM complicated by ischemic stroke across all 4 CPM interventions. The network meta-analysis of the 4 direct comparisons revealed that TXL combined with CT had the highest number of studies (5^[9–12,18]^) and the largest overall sample size (443 cases) compared to CT, as depicted in Figure 6.

**Figure 11.**
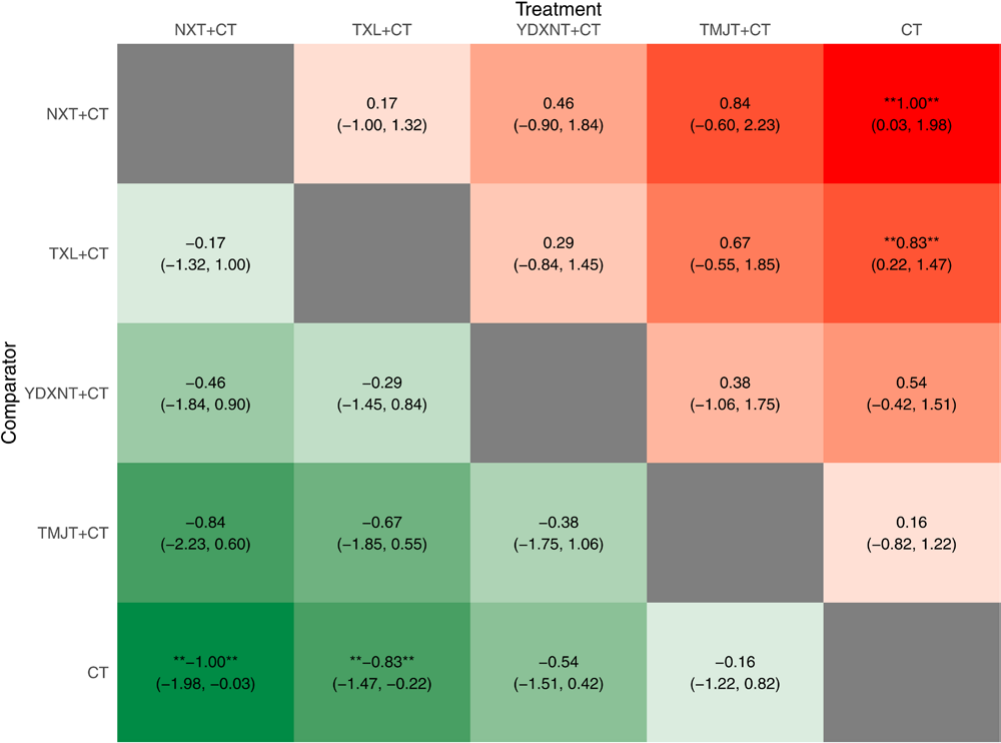
Heat map of LDL.

The results demonstrated that NXT + CT and TXL + CT significantly improved LDL levels compared to CT, with statistically significant differences (*P* < .05). However, there was no significant difference observed in the improvement of LDL levels between the other 2 CPM interventions combined with CT and CT (*P* > .05), as depicted in Figure 11.

**Figure 12.**
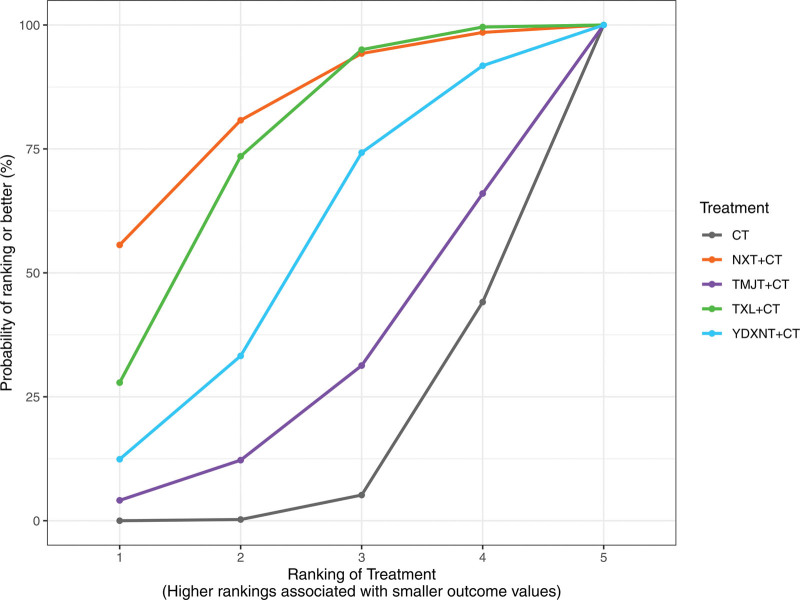
SUCRA ranking diagram of LDL.

Based on the surface under the SUCRA probabilities ranking, the interventions were ranked as follows: NXT + CT (SUCRA = 82.27%) > TXL + CT (SUCRA = 73.99%) > YDXNT + CT (SUCRA = 52.96%) > TMJT + CT (SUCRA = 28.34%) > CT alone (SUCRA = 12.43%). These results suggest that NXT + CT may be the optimal intervention for improving LDL levels in patients with T2DM complicated by ischemic stroke, as shown in Figure 12.

### 3.8. Adverse events

A total of 4 RCTs^[13,16,19,20]^ provided information on the occurrence of adverse reactions. The incidence of adverse reactions in the treatment group was 8.4%, which was significantly lower than the 17.4% observed in the control group (χ^2^ = 21.7, *P* < .05). Gastrointestinal reactions accounted for 56.67% of all reported adverse events, rash accounted for 30.67%, and abnormal liver function accounted for 8.00%. Upon thorough examination of the relevant literature, it was noted that all patients were taking statin drugs, and their symptoms resolved spontaneously after discontinuation of the medication. Please refer to Table 3 for additional details.

**Table 3:** Adverse reaction occurrence.

Group	Number	Dizziness	insomnia	Gingival bleeding	Gastrointestinal reactions	Skin Rash	Hepatic dysfunction	Adverse reaction occurrence rate
Treatment group	630	1	0	0	31	19	2	8.4%*
Treatment group	557	2	2	2	54	27	10	17.4%

*Note*: Compared to the control group.

* *P*<.05.

### 3.9. Medication rule

In this study, the 4 types of CPM interventions included in the analysis were categorized based on their therapeutic effects. These categories are as follows: (1) blood-activating herbs: this category includes 16 herbs, such as Chishao (Radix Paeoniae Rubra), Danshen (Radix Salviae Miltiorrhizae), Danggui (Radix Angelicae Sinensis), Chuanxiong (Rhizoma Ligustici Chuanxiong), Taoren (Semen Persicae), Honghua (Flos Carthami), Jixueteng (Caulis Spatholobi), Niuxi (Radix Achyranthis Bidentatae), Dilong (Pheretima Aspergillum), Quanxie (Scorpio), Shuizhi (Hirudo), Wugong (Scolopendra), Tubiechong (Eupolyphaga seu Steleophaga), Yinxiangye (Ginkgo Folium), Dengzhanxi Xin (Nardostachys Jatamansi), and Sanqi (Radix Notoginseng). This category of herbs had the highest frequency of use in the treatment of T2DM complicated by ischemic stroke. (2) Qi-regulating herbs: this category includes 3 herbs, namely Ruxiang (Boswellia Resin), Jiangxiang (Dalbergia Odorifera), and Moyao (Commiphora Myrrha). (3) Phlegm-dispersing and turbidity-eliminating herbs: This category consists of 5 herbs, including Huanglian (Coptidis Rhizoma), Jiaogulan (Gynostemma Pentaphyllum), Cangzhu (Atractylodes Rhizoma), Dongkuizi Guo (Malva Verticillata Fruit), and Shanzi (Fructus Crataegi). (4) Spleen-tonifying and qi-strengthening herbs: This category includes 5 herbs, namely Huangqi (Radix Astragali), Renshen (Radix Ginseng), Taizishen (Pseudostellariae Radix), Xuanshen (Scrophulariae Radix), and Shanyao (Rhizoma Dioscoreae). The results of the study suggest that the use of blood-activating herbs is the most frequent among the 4 categories, indicating their importance in improving blood lipid levels in patients with T2DM complicated by ischemic stroke.

## 4. Conclusion

Comorbidity, the prevalent pattern of disease occurrence in modern society, poses a significant threat to human well-being. Western medicine lacks comprehensive treatment approaches for multiple diseases that lack a direct causal relationship. Relying solely on targeted treatments for individual diseases is insufficient to meet the growing healthcare demands. CPM, with its unique theoretical system, plays a crucial role in disease treatment by addressing the fundamental causes of diseases. Therefore, it is imperative to embrace the essence of CPM theory and promote the complementary and coordinated development of CPM and Western medicine. In CPM, comorbidity refers to a group of diseases sharing similar pathogenesis. According to CPM, diseases arising from different causes, such as the “Five Movements and Six Qi” and the “Seven Emotions and Six Desires,” can lead to organ imbalances and dysfunctions, ultimately resulting in different diseases with similar underlying mechanisms. CPM adheres to the principle of treating different diseases with similar approaches to address these conditions characterized by shared pathogenic mechanisms. CPM utilizes its unique theoretical advantages to elucidate the intrinsic connections among seemingly unrelated diseases. It emphasizes treating diseases at their roots and seeking the fundamental causes. Through this holistic approach, CPM addresses the underlying imbalances and dysfunctions that contribute to the simultaneous development of multiple diseases.

The prevalence of lipid metabolism disorders in China has shown a steady increase, particularly affecting younger individuals.^[21]^ Despite dyslipidemia being an independent risk factor for various diseases, including cardiovascular diseases, its detrimental effects are often overlooked by many individuals. Lipid metabolism plays a critical role in the development of diabetes, and research indicates that lipids serve as the primary source of bioavailable heme iron. Excessive lipid accumulation and dyslipidemia lead to significant heme iron generation, resulting in oxidative damage and the onset of insulin resistance.^[22]^ Moreover, excessive carbohydrate consumption stimulates the secretion of acetyl-CoA, leading to increased production of oxygen free radicals and further exacerbation of insulin resistance, diabetes, and its complications.^[23]^ Additionally, elevated levels of total cholesterol and cholesterol deposition in the arterial intima contribute to increased blood viscosity, endothelial damage, the progression of atherosclerosis, and an augmented risk of thrombosis formation.^[24]^ Therefore, blood lipids serve as a connection between these 2 diseases, linking them through shared pathophysiological mechanisms.

The ancient text “Ling Shu Jing” acknowledges the presence of “fat, grease, and flesh” as inherent components of the human body rather than pathological states. CPM attributes lipid metabolism disorders to the overconsumption of fatty and sweet foods, which damage the qi of the spleen and stomach, leading to impaired transportation and transformation. This imbalance disrupts the distribution of water and grains in the body, resulting in excessive accumulation. Qi circulation is obstructed, blood circulation weakens, blood stasis occurs, and dampness transforms into phlegm. From the CPM perspective, “phlegm dampness” and “blood stasis” denote abnormal states of blood lipids.^[25]^ In this context, “fat and “grease are considered pathological manifestations, and the crucial factor lies in the accumulation of “phlegm dampness and “blood stasis within the body. Therefore, CPM diagnosis and treatment go beyond addressing the biochemical abnormalities of blood lipids and aim to target the underlying causes of the disease. Academician Tong Xiaolin has reclassified and staged diabetes in CPM, categorizing T2DM as “spleen and pancreas dampness.”^[26]^ This classification highlights the association with spleen deficiency and impaired transportation and transformation. As described in “Su Wen, Qi Bing Lun,” individuals who are overweight and prone to consuming sweet and fatty foods may experience internal heat and abdominal fullness, leading to excessive qi and thirst. Improper diet can impair the spleen and stomach, resulting in the generation of phlegm dampness, obstruction of qi circulation, weak blood circulation, blood stasis, and heat production. This leads to deficiencies in both qi and yin, representing the pathogenesis of spleen and pancreas dampness. Ischemic stroke, known as “zhong feng” in CPM, is characterized by insufficient blood supply to the brain, resulting in continuous ischemia, hypoxia, and eventual necrosis of brain tissue. The text “Su Wen” states that individuals who are overweight and affluent are more susceptible to this condition. “Yi Fang Kao” identifies “phlegm turbidity” and “blood stasis” as the main pathogenic factors and pathological manifestations in cases of stroke where the hands and feet are nonfunctional and the condition does not improve over time. In summary, the comorbidity between T2DM and ischemic stroke can be elucidated through the CPM perspective of “phlegm and blood stasis.”

The study’s major strength lies in its comprehensive comparison of the clinical efficacy of various Chinese patent medicines combined with CT in T2DM patients with ischemic stroke, with a particular focus on TC, TG, and LDL. The results demonstrate the following key findings: (1) regarding TC levels, the combination of YDXNT + CT exhibited superior effectiveness compared to the combination of NXT and CT, while the combination of TXL and CT showed the least impact. Notably, CT alone had the weakest effect. These findings suggest that the combination of YDXNT and CT may be the optimal intervention for reducing TC levels. (2) Concerning TG levels, the combination of TXL and CT demonstrated the most significant therapeutic effect among the 4 Chinese patent medicines, while the other 3 medicines exhibited insignificant effects. (3) In terms of improving LDL levels, the combination of NXT and CT outperformed the combination of TXL and CT, while the other 2 Chinese patent medicines showed negligible effects. (4) When compared to CT alone, the combination of Chinese patent medicines with CT resulted in a lower incidence of adverse reactions in T2DM patients with ischemic stroke. These findings provide valuable insights into the clinical efficacy of different treatment approaches and suggest potential options for improving lipid metabolism in T2DM patients with ischemic stroke.

YDXNT is composed of several herbal ingredients, including Ginkgo biloba leaf, *Salvia miltiorrhiza* (Danshen), *Panax notoginseng* (Sanqi), *Ligusticum chuanxiong* (Chuanxiong), *Centella asiatica* (Gotu Kola), and *Crataegus pinnatifida* (Hawthorn). It exhibits effects in promoting blood circulation, resolving stasis, and reducing turbidity and lipid levels. Ginkgo biloba leaf is commonly used in the treatment of diabetes, its complications, and cardiovascular diseases.^[27]^ Modern pharmacological studies have shown that Ginkgo biloba leaf effectively inhibits platelet aggregation, improves blood perfusion, and reduces levels of TC, TG, and LDL cholesterol in the blood.^[28]^ Danshen, a widely used herb for promoting blood circulation and resolving stasis, contains 2 main components: phenolic acids and tanshinones. Phenolic acids effectively reduce vascular permeability, inhibit platelet aggregation, dilate arterial vessels, improve cerebral circulation and cognitive impairment, and protect and repair damaged brain tissue. Tanshinones possess anticoagulant effects, improve atherosclerosis, and enhance cardiovascular and neurological activity.^[29]^ Wu Zhihuan et al^[30]^ conducted experiments and found that after intervention with Sanqi-Danshen in a high-fat model group of mice, the blood lipid and blood glucose levels returned to normal levels. Ligusticum chuanxiong, through regulating hemodynamics, inhibiting platelet aggregation, and repairing damaged vascular endothelial cells, can improve blood lipid levels in stroke patients and significantly enhance their motor function.^[31]^ The combination of *Centella asiatica* and *Crataegus pinnatifida*, as representative herbs for reducing turbidity and lowering lipid levels, can significantly improve blood lipid levels. This may be achieved by inhibiting reactive oxygen species and enhancing the body’s antioxidant stress response, which is a common mechanism in the treatment of ischemic stroke and T2DM.^[32,33]^ NXT and TXL share many similarities in their herbal compositions. NXT is based on the classic traditional Chinese medicine formula Bu Yang Huan Wu Tang, with the addition of Danshen, Scolopendra subspinipes mutilans (centipede), and Panax ginseng (Ginseng). These additions contribute to promoting blood circulation, resolving stasis, and tonifying qi. Bu Yang Huan Wu Tang is commonly used in traditional Chinese medicine for treating stroke (stagnation of blood obstructing collaterals). Modern research has demonstrated that Bu Yang Huan Wu Tang regulates lipid metabolism, inflammatory responses, oxidative stress, and improves insulin resistance through various signaling pathways, such as FOXO, TNF, HIF-1, and PI3K-Akt, thereby treating hyperlipidemia.^[34]^ The high-dose fibrinolytic protein in the extract of Scolopendra subspinipes mutilans inhibits the activity of Caspase-3 (a protease) and has anticoagulant activity, inhibiting arterial and meridian thrombosis formation.^[35]^ Sun Wei et al found that after intervention with Ginseng extract in a high-fat mouse model, ginsenosides showed significant lipid-lowering effects, possibly by altering the structure and quantity of intestinal flora.^[36]^ Furthermore, NXT can reduce blood-brain barrier permeability, protect vascular endothelial cells, decrease necrosis of ischemic brain tissue, inhibit the activity of inflammatory factors such as IL-1β, and alleviate brain ischemia-reperfusion injury.^[37,38]^ TXL comprises *Astragalus membranaceus* (Huangqi), *Salvia miltiorrhiza* (Danshen), *Carthamus tinctorius* (Honghua), and *Cistanche deserticola* (Rou Cong Rong). The overall formula promotes blood circulation, resolves stasis, invigorates the spleen, and benefits qi. Li Jianping et al constructed an insulin-resistant mouse model and administered the Danshen-Honghua herbal pair, finding significant improvement in abnormal TC and TG levels in insulin-resistant mice and repair of damaged pancreatic β-cells.^[39]^ The mechanisms of action of Huangqi polysaccharides are extensive, including improving leptin sensitivity to enhance lipid metabolism in hyperlipidemic rats, reducing lipid accumulation, promoting lipid decomposition, and increasing insulin sensitivity to repair pancreatic β-cell dysfunction and reduce fasting blood glucose levels in T2DM rats, making them applicable in the treatment of circulatory and nervous system disorders.^[40]^ Studies have revealed that TXL can repair damaged neurons in brain ischemia-reperfusion injury in mice, with the area of cerebral infarction gradually decreasing with increasing dosage.^[41]^

This study offers valuable guidance for clinicians in the selection of medications to treat blood lipid disorders in patients with T2DM complicated by ischemic stroke. However, it is crucial to acknowledge several limitations that could affect the reliability and generalizability of the findings. These limitations include: (1) low methodological quality of included literature: the study acknowledges the varying overall quality of the literature utilized in this analysis, potentially impacting the reliability of the findings and warranting caution when interpreting the results. (2) Variations in intervention measures: Differences exist in the intervention measures employed across different RCTs, encompassing variations in drug frequency, dosage, and treatment duration. These discrepancies introduce heterogeneity and may compromise the comparability of the results. (3) Lack of large-scale multicenter clinical trials: The absence of large-scale multicenter clinical trials restricts the generalizability of the findings. More robust evidence from such trials is necessary to enhance the validity and applicability of the study’s conclusions. (4) Absence of pairwise comparisons of CPMs: The absence of clinical trials directly comparing different CPMs creates an incomplete evidence network. This shortcoming may impact the overall validity and stability of the study’s results, underscoring the need for further research to address this gap.

## 5. Conclusion

To address these limitations, future research should prioritize conducting high-quality studies with standardized interventions and larger sample sizes. This approach would enhance the understanding and applicability of the findings in clinical practice. In conclusion, the integration of CPM principles, such as “activating blood circulation” and “reducing turbidity,” as a complementary approach to conventional Western medicine treatment has demonstrated promising results in regulating blood lipid levels, improving prognosis in ischemic stroke patients, and managing T2DM and its complications. Future studies should place emphasis on conducting pairwise comparisons of different Chinese patent medicines to enhance the quality and precision of the evidence. This would enable clinicians to make more informed decisions based on the individual characteristics of patients. By expanding the knowledge base and refining treatment approaches, the optimization of the integration of CPM and Western medicine can benefit patients.

## Author contributions

**Conceptualization:** Yanming Xie.

**Data curation:** Zhenkai Lu.

**Formal analysis:** Zhenkai Lu.

**Funding acquisition:** Yanming Xie.

**Methodology:** Zhenkai Lu, Xuming Zhang.

**Resources:** Yanming Xie.

**Software:** Xuming Zhang.

**Validation:** Zhenkai Lu.
